# On Amputation

**Published:** 1810-04

**Authors:** 


### *To the Editors of the Medical and Physical Journal.*

Gentlemen,

In your Journal of the present month, there are some observations upon cases which require amputation, where no tourniquet can be applied from its nearness to the trunk, and where there is sufficient room to operate without opening the joint. Thus your correspondent, in the amputation of the shoulder-joint, as the first step, tied the artery in the axilla: I will take the liberty of putting the question to him, whether a sufficient and steady pressure cannot be made upon the artery above the clavicle, so as effectually to stop the current of blood in the axillary artery; which if it can be done, is certainly far preferable, considering the hazard of such an operation, as tying the artery in the axilla, situate in a deep cavity, surrounded with nerves and veins; as well as the consideration of putting the patient to unnecessary pain. That a sufficient pressure can be made on the artery in the groin, so as effectually to stop the current of blood in the femoral artery, I am well convinced, from the numerous operations for the femoral and popliteal aneurisms which I have witnessed.

I am, &c.

A YOUNG SURGEON.

*March* 7, 1810.

